# Quantitative Trait Transcripts Mapping Coupled with Expression Quantitative Trait Loci Mapping Reveal the Molecular Network Regulating the Apetalous Characteristic in *Brassica napus* L.

**DOI:** 10.3389/fpls.2018.00089

**Published:** 2018-02-01

**Authors:** Kunjiang Yu, Xiaodong Wang, Feng Chen, Qi Peng, Song Chen, Hongge Li, Wei Zhang, Sanxiong Fu, Maolong Hu, Weihua Long, Pu Chu, Rongzhan Guan, Jiefu Zhang

**Affiliations:** ^1^Key Laboratory of Cotton and Rapeseed, Ministry of Agriculture, Institute of Industrial Crops, Jiangsu Academy of Agricultural Sciences, Nanjing, China; ^2^State Key Laboratory of Crop Genetics and Germplasm Enhancement, Nanjing Agricultural University, Nanjing, China; ^3^College of Agriculture, Guizhou University, Guiyang, China

**Keywords:** *Brassica napus* L., apetalous, quantitative trait transcript, expression QTL, regulatory network

## Abstract

The apetalous trait of rapeseed (*Brassica napus*, AACC, 2*n* = 38) is important for breeding an ideal high-yield rapeseed with superior klendusity to *Sclerotinia sclerotiorum*. Currently, the molecular mechanism underlying the apetalous trait of rapeseed is unclear. In this study, 14 petal regulators genes were chosen as target genes (TGs), and the expression patterns of the 14 TGs in the AH population, containing 189 recombinant inbred lines derived from a cross between apetalous “APL01” and normal “Holly,” were analyzed in two environments using qRT-PCR. Phenotypic data of petalous degree (PDgr) in the AH population were obtained from the two environments. Both quantitative trait transcript (QTT)-association mapping and expression QTL (eQTL) analyses of TGs expression levels were performed to reveal regulatory relationships among TGs and PDgr. QTT mapping for PDgr determined that *PLURIPETALA* (*PLP*) was the major negative QTT associated with PDgr in both environments, suggesting that *PLP* negatively regulates the petal development of line “APL01.” The QTT mapping of *PLP* expression levels showed that *CHROMATIN-REMODELING PROTEIN 11* (*CHR11*) was positively associated with *PLP* expression, indicating that *CHR11* acts as a positive regulator of *PLP* expression. Similarly, QTT mapping for the remaining TGs identified 38 QTTs, associated with 13 TGs, and 31 QTTs, associated with 10 TGs, respectively, in the first and second environments. Additionally, eQTL analyses of TG expression levels showed that 12 and 11 unconditional eQTLs were detected in the first and second environment, respectively. Based on the QTTs and unconditional eQTLs detected, we presented a hypothetical molecular regulatory network in which 14 petal regulators potentially regulated the apetalous trait in “APL01” through the *CHR11-PLP* pathway. *PLP* acts directly as the terminal signal integrator negatively regulating petal development in the *CHR11-PLP* pathway. These findings will aid in the understanding the molecular mechanism underlying the apetalous trait of rapeseed.

## Introduction

Flowers of angiosperms are typically composed of four organ types inclined to four floral whorls. From the outside of the flower to the center, these organs are orderly sepals, petals, stamens, and carpels (the subunits of the gynoecium). Over the last 20 years, the molecular mechanism of flower development have been adequately elucidated in several angiosperm species, such as *Arabidopsis thaliana, Antirrhinum majus, Petunia hybrid*, and *Oryza sativa* (Schwarz-Sommer et al., 1990; Bowman et al., 1991; van der Krol and Chua, 1993; Li et al., 2011; Hirano et al., 2014). Recently, the genetics of flower development in *Ranunculales* were also decoded successfully (Damerval and Becker, 2017). The “ABC model” as the basic model explaining both floral patterning and floral organ identity has been endlessly enriched by works in several eudicot species (Pelaz et al., 2000; Jack, 2001; Theissen and Saedler, 2001). Currently, the “ABCE model,” as the most detailed floral model, is guiding investigations that will aid in understanding the origin and diversification of angiosperm flowers.

Petal initiation, a key unit of flower development, is crucial in revealing the evolutionary history of flowering plants. According to the “floral quarter model,” A class (*APETALA 1, AP1*), B class (*APETALA3* and *PISTILLALA, AP3* and *PI*, respectively), and E class (*SEPALLALA 1/2/3, SEP1/2/3*) genes are simultaneously required for petal identity in *Arabidopsis* (Theissen and Saedler, 2001; Ditta et al., 2004). Molecular evolutionary studies indicated that B class genes underwent two vital duplication and divergence events, in which the first event generated the *PI* and paleo*AP3* lineages, while the second event generated eu*AP3* and *TM6* lineages (Kramer et al., 1998; Kim et al., 2004). Both paleo*AP3* and *TM6* have the same paleo*AP3* motif regulating stamen development, but they are not involved in petal development (Kramer et al., 1998; Kim et al., 2004; Rijpkema et al., 2006). Eu*AP3* contains the eu*AP3* motif required for development of both petals and stamens (Vandenbussche et al., 2004; de Martino et al., 2006; Rijpkema et al., 2006; Drea et al., 2007; Kramer et al., 2007; Hileman and Irish, 2009). Strangely, although there are both eu*AP3* and *TM6* in most eudicots, there is only eu*AP3* in *Arabidopsis* and snapdragon (Lamb and Irish, 2003; Vandenbussche et al., 2004). In addition to B class genes, there are a number of genes involved in petal development in *Arabidopsis*, many of which function upstream or downstream of ABE class genes (Kaufmann et al., 2009, 2010; Wuest et al., 2012). However, the locations of some genes in the regulatory network of petal development are unclear, such as *PLURIPETALA* (*PLP*) (Running et al., 2004) and *CHROMATIN-REMODELING PROTEIN 11*(*CHR11*) (Smaczniak et al., 2012).

Apetalous rapeseed, which is a novel floral mutant in which the whorl organs are perfectly developed separate from the petals, has advantages of low-energy consumption, high photosynthetic efficiency and superior klendusity to *Sclerotinia sclerotiorum* (Chapman et al., 1984; Yates and Steven, 1987; Morrall, 1996; Jamaux and Spire, 1999). Thus, apetalous rapeseed is considered the ideotype of high-yield rapeseed (Mendham and Rao, 1991; Rao et al., 1991), and it has attracted the attention of botanists and breeders since its appearance. Currently, the molecular mechanism underlying the apetalous characteristic of rapeseed is poorly known because of the lack of stable apetalous mutants and the complexity of polygenic inheritance (Kelly et al., 1995; Fray et al., 1997; Wang et al., 2015; Yu et al., 2016). The apetalous characteristic of rapeseed is mainly governed by recessive genes, usually by two to four loci (Kelly et al., 1995), and several quantitative trait loci (QTLs) regulating petal development on chromosomes A3, A4, A5, A6, A9, C4, and C8 have been identified (Fray et al., 1997; Wang et al., 2015). A deficiency in eu*AP3* expression may give rise to the apetalous characteristic, while the paleo*AP3* expression ensures stamen development in *Brassica napus* (Zhang et al., 2011). This theory, coupled with the “ABCE model,” predicts that sepals of apetalous rapeseed should increase, but the number of sepals is actually normal (Zhang et al., 2011). This indicates that the molecular mechanism controlling the apetalous characteristic of rapeseed is more complex than initially believed.

In our previous study (Wang et al., 2015), nine QTLs associated with petalous degree (PDgr) have been detected on chromosomes A3, A5, A6, A9, and C8 in the AH population, containing 189 recombinant inbred lines derived from a cross between an apetalous line “APL01” and a normal petalled variety “Holly.” Interestingly, three QTLs, *qPD.A9-2, qPD.C8-2*, and *qPD.C8-3*, are stably expressed in multiple environments (Wang et al., 2015). In another study (Yu et al., 2016), genome-wide transcriptomic analyses of the apetalous line “APL01” and another normally petalled line “PL01” both derived from the F_6_ generation of crosses between apetalous “Apetalous No. 1” and normal petalous “Zhongshuang No. 4” rapeseed have been performed. Further analysis suggested that a large number of genes involved in protein biosynthesis were differentially expressed at the key stage of petal primordium initiation in “APL01” compared with in “PL01,” and 36 petal regulators implicated in the apetalous trait of line APL01 were identified (Yu et al., 2016). Interestingly, the 36 petal regulators were outside of the confidence intervals (CIs) of nine QTLs regulating PDgr, implying that these genes maybe function at the downstream of the QTLs (Yu et al., 2016). However, it's worth noting that mutants of the 36 petal regulators result in defective floral phenotypes other than abnormal petals in *Arabidopsis*, such as (*PLP*) (Running et al., 2004) and (*CHR11*) (Smaczniak et al., 2012). For the aptelous characteristic of rapeseed, these genes collaboratively participate in the regulation of petal development, leading to the unique floral phenotype of “APL01.” However, the specifics of this collaborative participation are unclear. Thus, it is necessary to analyze relationships among petal regulators and PDgr using multiple approaches.

A quantitative trait transcript (QTT) analysis is a mixed linear model approach of association mapping of a transcriptome (Zhang et al., 2015). So far, QTT has been applied to detect the transcripts associated with complex traits in mice (Zhang et al., 2015), rice (Zhou et al., 2016), and human (Chen et al., 2016) populations, and it has efficiently identified the genetic effects of individual loci, and epistatic interactions of pair-wise loci or gene-by-gene (G×G) (Zhang et al., 2015; Chen et al., 2016; Zhou et al., 2016). Expression QTL (eQTL) analysis based on linkage mapping is an approach to determining gene expression levels (Jansen and Nap, 2001). This approach can identify the genetic determinants of gene expression levels and has been successfully used to investigate gene regulatory pathways in plants (DeCook et al., 2005; Jordan et al., 2007; Yin et al., 2010; Wang et al., 2014), animals (Sun et al., 2003; Ghazalpour et al., 2006; Li et al., 2006), and humans (Cheung et al., 2003; Göring et al., 2007; Battle and Montgomery, 2014). Conditional QTL mapping is a method that can exclude the contribution of a causal trait to the variation of the resultant trait (Zhu, 1995). Unconditional QTL mapping coupled with conditional QTL analysis could dissect the genetic relationships between two traits at the QTL level, and then it has been broadly applied to exploring the relationships between QTLs and the corresponding conditional traits (Zhao et al., 2006; Cui et al., 2011; Zhang et al., 2013).

In this study, we analyzed the expression levels of the 36 petal regulators genes and 1 candidate gene *CG1* (*BnaC08g10840D*), underlying the CI of the major QTL *qPD.C8-2* in “APL01,” “PL01,” and “Holly” by using qRT-PCR. The comparative analyses indicated that both 13 petal regulators genes and *CG1* showed the same dynamic expression levels between “APL01” and “PL01” as between “APL01” and “Holly.” Thus, the 14 genes were chosen as target genes (TGs) for quantitative reverse transcription-PCR (qRT-PCR) analyses. The expression patterns of the 14 TGs in the AH population were analyzed in two environments using qRT-PCR. Phenotypic data of PDgr in the AH population were obtained from the two environments. Regulatory relationships among TGs and PDgr were discovered, genomic regions influencing TGs expression were identified, and molecular networks regulating the petal development of an apetalous line “APL01” were constructed as a result of QTT-association mapping coupled with eQTL analyses of TGs expression levels.

## Materials and methods

### Plant materials

“APL01” and “PL01” was selected from the F_6_ generation of crosses between apetalous (“Apetalous No. 1”) and normal petalous (“Zhongshuang No. 4”) rapeseed in 1998. “Apetalous No. 1” had been developed from the F_8_ generation of crosses between a Chinese rapeseed cultivar with smaller petals (SP103) and *B. rapa* variety with a lower PDgr (LP153). “Zhongshuang No. 4” was bred at the Oil Crops Research Institute of the Chinese Academy of Agricultural Sciences, Wuhan, China. The AH population, containing 189 recombinant inbred lines (RILs), was derived from a cross between an apetalous line “APL01” and a normally petalled variety “Holly.” The genotype “Holly” is a completely petalled variety. The AH population was planted in two different districts, Lishui County (coded 2015a) and Xuanwu District (coded 2015b), in Nanjing of Jiangsu Province for one year (September-May of 2014-2015) with good field management measures. The subsequent works were independently performed in both environments.

### Collection of samples, and evaluation of PDgr

According to our previous study (Yu et al., 2016) and with early flower development studies in *B. napus* (Polowick and Sawhney, 1986) and in *Arabidopsis* (Smyth et al., 1990), the petal primordia appear in the second whorl later in stage 5, but the petal primordia begin growing rapidly at the start of stage 9 in *B. napus*. The length of buds in stage 10 is at least double that of buds in stage 9. To minimize the sampling error, young inflorescences only containing buds at stages 1 to 9 were gathered for the subsequent works after removing stage 10 to 12 buds during flower bud development. At least five young inflorescences derived from five plants in each RIL of the AH population were collected in each environment. A total of two biological samples were collected in each RIL of the AH population. For lines “APL01,” “PL01,” and “Holly,” three biological samples of each line were separately collected. The actual and theoretic numbers of flower petals were recorded in each RIL at early blooming stage. The evaluation of PDgr was carried out as described in our previous study (Wang et al., 2015).

### Total RNA exaction, cDNA synthesis, and qRT-PCR assay

Total RNA was isolated using MagaZorb® Total RNA Mini-Prep Kit (Promega, Madison, WI, USA). RNA degradation and contamination were checked on 1% agarose gels. The RNA concentration was measured using the Q3000® Micro-Ultraviolet Spectrophotometer (Quawell, Sunnyvale, CA, USA). First-strand cDNAs were synthesized in a final volume of 20 μL containing 4 μL of 5 × PrimeScript RT Master Mix (Perfect Real Time), ≤1 μg of total RNA, and <16 μL of RNase Free dH_2_O using PrimeScript™ RT Master Mix (Perfect Real Time) (TaKaRa, Da Lian, China). Sequences of TGs and paralogs were obtained from the *B. napus* genome database (http://www.genoscope.cns.fr/brassicanapus/) (Chalhoub et al., 2014). Primers for the qRT-PCR assay were designed using Primer 5 software and synthesized by Sangon Biotech (Shanghai, China) (Table S1). The rapeseed *ACTIN* (*BnaA05g21350D*) gene was chosen as the endogenous reference gene to examine the sample-to-sample variation in the amount of cDNA. Each reaction (20 μL) contained 10 μL of 2 × SYBR Premix Ex Taq (Tli RNaseH Plus), 0.8 μL of 10 μM gene-specific primers, 0.4 μL of 50 × ROX Reference Dye II, <100 ng of first-strand cDNAs, and <8.8 μL of RNase Free dH_2_O according to SYBR® Premix Ex Taq™ (Tli RNaseH Plus) (TaKaRa). The three-step PCR (95°C for 30 s, followed by 40 cycles of 95°C for 5 s, 55°C for 30 s, and 72°C for 30 s) was performed with the ABI PRISM 7500 Real-Time PCR System (Applied Biosystems, Foster, CA, USA). For the qRT-PCR assay on “APL01” vs. “PL01,” or “Holly,” the later was chosen as the sample for reference. For the qRT-PCR assay in the AH population, RIL43 was chosen as the reference sample. Triplicate replicates for each qRT-PCR assay were performed independently.

### Data collection, identification of TGs, and drafting of standard curves

PCR cycles (C_t_) for all genes were determined in each amplification reaction after removing the reactions with nonspecific and/or unrepeatable amplifications. The relative expression levels of the genes in different samples were calculated using 2^−ΔΔCt^ method (Livak and Schmittgen, 2001), defined as: ΔΔCt = (C_t, target_−C_t, actin_)_genotype_−(C_t, target_−C_t, actin_)_calibrator_, in which “genotype” indicates the target sample and “calibrator” indicates the reference sample. In our previous study, 36 petal regulators and 1 candidate gene were identified as differentially expressed genes in line APL01 compared with line PL01 (Yu et al., 2016). In this study, whether the differences in these genes' expression levels between “APL01” and “PL01” or “Holly” are significant depends on the *P*-value estimated using SPSS Statistics 19.0 software (IBM, Armonk, NY, USA) (non-paired *t*-test, *P* < 0.05). Genes showing the same expression patterns between “APL01” and “PL01” as between “APL01” and “Holly” were regarded as TGs for the subsequent analyses. Standard cDNA was diluted 10, 15, 20, 25, 30, and 35 times before the qRT-PCR analysis. The cDNA's dilution ratio is the independent variable of the standard curve, while the C_t_ values of the TGs and *ACTIN* are the dependent variables. Standard curves of TGs were drawn using Sigma Plot 12.5 software (Systat Software Inc., San Jose, CA, USA). TG expression levels in the AH population were used for QTT mapping and eQTL analysis after removing low quality data. The non-specific PCR amplification of *ACTIN* in each cDNA sample was regarded as the standard for estimating low quality data because the *ACTIN* primer pair consisted of cross-intron primers. To further evaluate the reliability of qRT-PCR data, all of the TG expression data was normalized using the following formula:

$$\begin{array}{l} \text{y}=\frac{q-a}{SD} \end{array}$$

in which “*y*” represents the normalized expression data of TG, “*q*” represents the TG expression level (2^−ΔΔCt^) in each RIL of the AH population, “*a*” indicates the average of the TG expression levels in the AH population, and “*SD*” is the standard deviation of the TG expression levels in the AH population.

The scatter plot diagram of the normalized expression data of TGs was drawn using Adobe Photoshop CS6 v13.0 software (Adobe Systems Inc., San Jose, CA, USA). The qualified qRT-PCR data should be located in the interval ranging from −2 to 2.

### Correlation analysis, and QTT-association mapping for PDgr and TGs

The correlations of PDgr with the TG expression levels in the AH population were assessed using SPSS Statistics 19.0 software (Bivariate correlation, Pearson, *P* < 0.05). QTT-association mapping of PDgr and TGs expression levels in the AH population was performed based on a mixed linear model approach using the QTT functional module of the QTXNetwork software (Zhang et al., 2015). For the QTT analysis of PDgr, the 14 TGs expression levels were the genotypic data, while PDgr was the phenotypic data in each assay. The transcript locus regulating PDgr was called QTT to correspond with the TG. Subsequently, QTT mapping of TGs were performed, and the expression levels of the TGs regulating PDgr served as the phenotypic data, while the remaining TG expression levels served as the genotypic data. QTT regulating TG expression level was called tQTT to correspond with the TG. To the same analogy, QTT-association mapping of the tQTTs (TGs) regulating the corresponding TG expression levels was performed in sequence. The mapping order and permutation time were set to 3 and 1000, respectively. The superior x-Ome prediction was also included. The *P* threshold for declaring a QTT (tQTT) significant was set as 0.05 (−*LogP* > 1.3). The normalized expression data of TG was used for QTT analysis. For mapping transcripts in homozygote population, the dependent variables (*y*_*kh*_) of the *k-th* subject in the *h-th* environment can be expressed by the following mixed linear model (Zhang et al., 2015):

$$\begin{array}{l} y_{kh}={\mu +e}_{h}+\underset{i}{\sum }q_{i}u_{ik}+\underset{i<j}{\sum }qq_{ij}u_{ijk}+\underset{i}{\sum }qe_{ih}u_{ikh}\\ +\underset{i<k}{\sum }qqe_{ijh}u_{ijkh}+\varepsilon_{kh} \end{array}$$

where *μ* represents the population mean; *e*_*h*_ represents the fixed effect of the *h-th* environment; *q*_*i*_ represents the *i-th* locus effect with coefficient *u*_*ik*_ (using expression values in QTT mapping); *qq*_*ij*_ represents the epistasis effect of locus *i* × locus *j* with coefficients *u*_*ijk*_ (using expression values *u*_*ik*_ × *u*_*jk*_ in QTT mapping); *qe*_*ih*_ represents the environment interaction effect of the *i-th* locus in the *h-th* environment with coefficient *u*_*ikh*_; *qqe*_*ijh*_ represents the epistasis × environment interaction effect of locus *i* × locus *j* in the *h-th* environment with coefficient *u*_*ijkh*_; and ε_*kh*_ represents the residual effect of the *k-th* individual in the *h-th* environment.

A QTT or tQTT with a heritability of at least 10% (*h*^2^ ≥ 10%) was considered the major QTT or tQTT, while QTT or tQTT that was detected repeatedly in the two environments was considered a stable QTT or tQTT. Both are considered as the key QTTs or tQTTs.

### Unconditional and conditional eQTL mapping of TGs

In our recent study (Wang et al., 2015), the AH genetic linkage map was constructed based on 2755 single-nucleotide polymorphism markers and 57 simple sequence repeats, and the QTLs for PDgr were been successfully detected. In this study, the TG expression levels in the AH population were regarded as phenotypic data for QTL linkage mapping, which was termed unconditional eQTL mapping. The software Windows QTL Cartographer 2.5 (Raleigh, NC, USA) was applied to perform the unconditional eQTL analysis (Wang et al., 2007). The composite interval mapping model was deployed for estimating putative eQTLs with additive effects (Zeng, 1994). The working speed and window size were set to 2, and 10 cM, respectively. The logarithm of odds threshold for detecting a significant eQTL ranged from 2.2 to 3.4 based on permutation test analyses (1,000 permutations, 5% overall error level) as described previously (Churchill and Doerge, 1994). Thus, the false discovery rate for eQTL analysis was 0.05. A conditional eQTL analysis was carried out as described by Zhu (1995). The key tQTTs were regarded as the conditional independent variables, and conditional expression levels (conditional dependent variables) of TGs were generated using the QGAstation software.

### Construction of the molecular network involved in petal development

Based on tQTTs and unconditional eQTLs, combined with our previous research (Wang et al., 2015; Yu et al., 2016), a regulatory network for the apetalous characteristic in “APL01” was constructed using Adobe Photoshop CS6 v13.0 software (Adobe Systems Inc).

## Results

### Identification of TGs, and TG expression levels in the AH population

**Figure 1:**
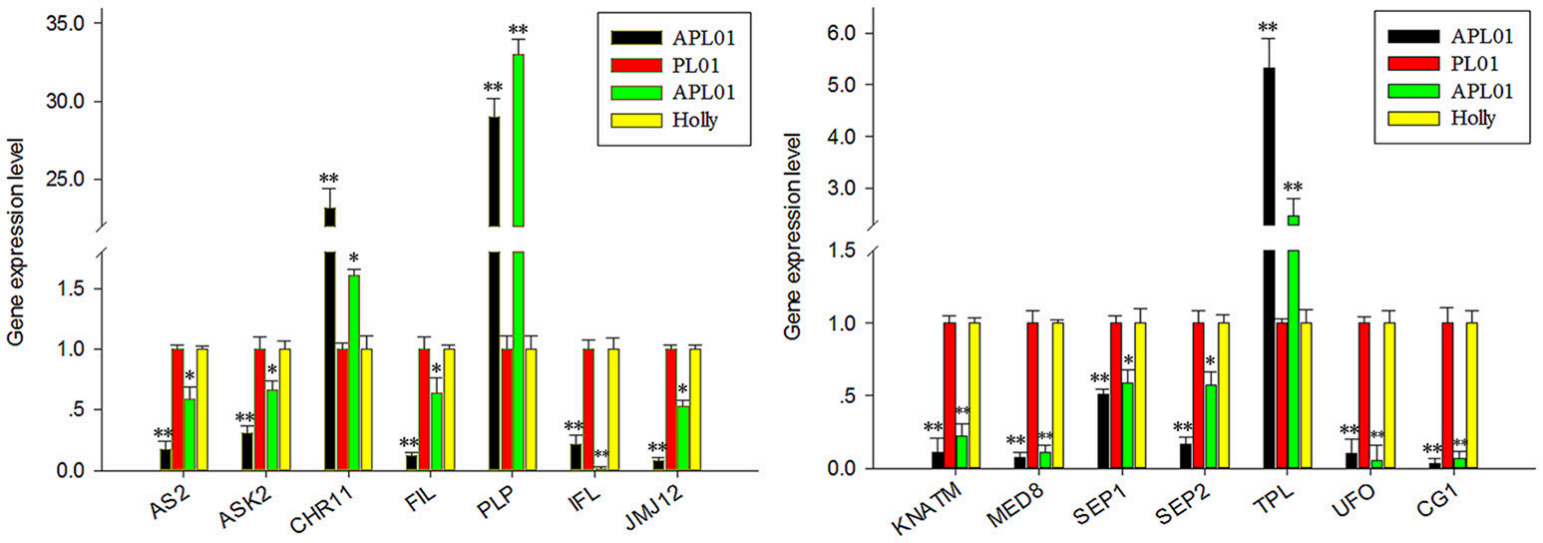
Verification of TG expression patterns by using qRT-PCR. Fourteen putative petal regulators showed the same expression patterns between “APL01” and “PL01” (black and red bars, respectively) as between “APL01” and “Holly” (green and yellow bars, respectively). Rapeseed *ACTIN* was chosen as the internal control to normalize the expression data. Data are the mean with standard error (SE) from three independent experiments. Single asterisk indicates that the difference is significant (non-paired *t*-test, *P* < 0.05), double asterisks indicate that the difference is extremely significant (non-paired *t*-test, *P* < 0.01).

In a previous study (Yu et al., 2016), 36 petal regulators and several candidate genes involved in the apetalous characteristic of line APL01 were obtained (Table S2). In this study, we determined that 13 petal regulators and 1 candidate gene *CG1* (candidate gene 1, *BnaC08g10840D*) showed the same expression patterns between “APL01” and “Holly” as between “APL01” and “PL01” as determined by qRT-PCR assays (Figure 1, Table S2). Thus, the 14 genes were regarded as TGs for the subsequent analyses. For these TGs, the expression levels of 3 genes increased at least 1.5-fold, while those of 11 decreased more than 1.6-fold in “APL01” compared with in “Holly” (Table S2).

**Figure 2:**
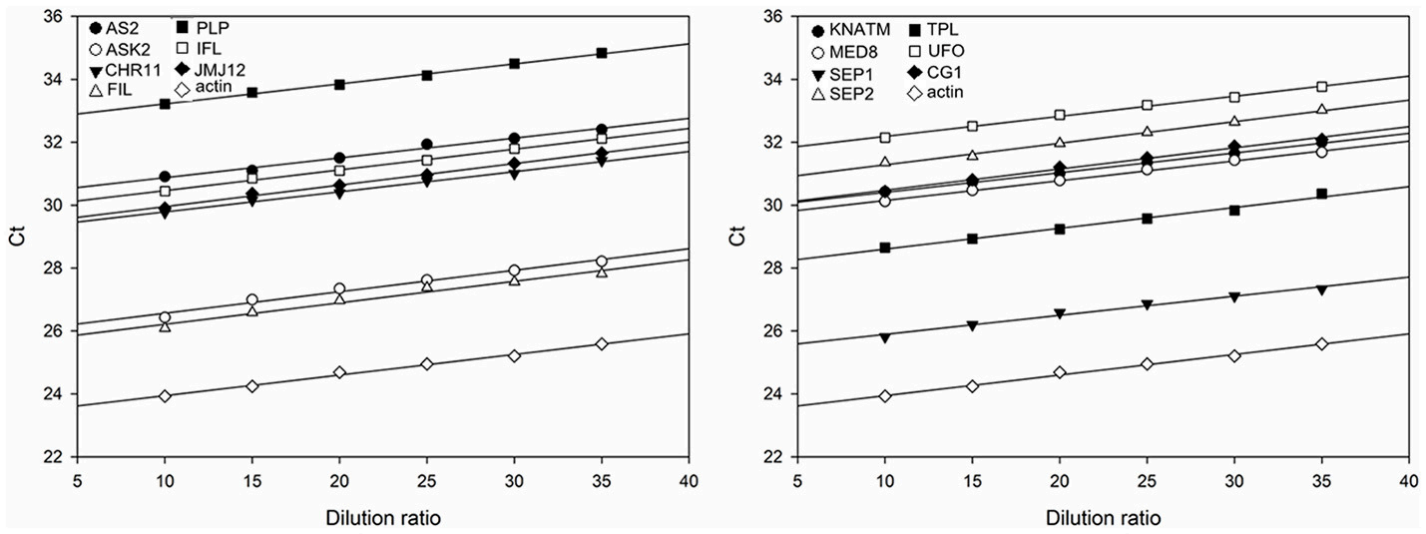
Standard curves for the amplification of 14 TGs and the endogenous reference gene *ACTIN*. The divisions on the horizontal axis represent the eight dilution ratio of standard cDNA, while the divisions on the vertical axis represent the threshold cycle values (C_t_) of the amplification. The amplification reactions of the TGs are described by the corresponding regression formulae (Table S3). The slope of the curves reflects the amplification efficiency of the corresponding TGs.

To estimate the relative expression levels of TGs, the rapeseed *ACTIN* was used as the endogenous reference gene to determine the sample-to-sample variation in the amount of cDNA. As shown in Figure 2, the slopes of the curves for each TG are almost to the same as that of *ACTIN*, indicating that the amplification efficiency was the same for the 14 TGs and *ACTIN* (Table S3). Subsequently, the expression levels of 14 TGs in the AH population were generated from the two environments using qRT-PCR. After removing low quality data, a high-quality dataset derived from 174 RILs was obtained for the next experiment. The scatter plot diagram of the normalized expression data of TGs suggested that most of data were located in the interval from −2 to 2 (Figure S1), indicating that qRT-PCR data used in this study was reliable.

### Correlation analysis

Correlation analyses between two biological replicates of TG expression within an environment determined that the Pearson correlation coefficient was at least 0.601, which means that qRT-PCR data was repeatable (Table S4). Correlation analysis of PDgr determined that the Pearson correlation coefficient was 0.806 between the two environments (Bivariate correlation, *P* = 2.01E-40) (Table 1), which suggests that there was a slight difference in PDgr between two environments. The expression levels of the TGs in the AH population, except for *CHROMATIN-REMODELING PROTEIN 11* (*CHR11*), *SEP1*, and *TOPLESS* (*TPL*), showed highly significant correlations between the two environments, and the Pearson correlation coefficients ranged from 0.266 to 0.925 (Table 1), indicating that the TGs' expression levels were differentially affected by different environments. Furthermore, random errors have an obvious effect on the difference in TG expression between the two environments probably.

**Table 1:** Correlation analyses of both TGs and PDgr in the AH population.

**Group A^a^**	**AS2_1^b^ vs. 2^c^**	**ASK2_1 vs. 2**	**CHR11_1 vs. 2**	**FIL_1 vs. 2**	**PLP_1 vs. 2**
*r*	0.876^**^	0.840^**^	0.029	0.468^**^	0.868^**^
	IFL_1 vs. 2	JMJ12_1 vs. 2	KNATM_1 vs. 2	MED8_1 vs. 2	SEP1_1 vs. 2
*r*	0.463^**^	0.925^**^	0.810^**^	0.266^**^	−0.012
	SEP2_1 vs. 2	TPL_1 vs. 2	UFO_1 vs. 2	CG1_1 vs. 2	PDgr_1 vs. 2
*r*	0.237^**^	0.028	0.564^**^	0.753^**^	0.806^**^
Group B^d^	AS2_1 vs. PDgr_1	ASK2_1 vs. PDgr_1	CHR11_1 vs. PDgr_1	FIL_1 vs. PDgr_1	PLP_1 vs. PDgr_1
*r*	−0.025	−0.076	−0.302^**^	−0.016	−0.442^**^
	IFL_1 vs. PDgr_1	JMJ12_1 vs. PDgr_1	KNATM_1 vs. PDgr_1	MED8_1 vs. PDgr_1	SEP1_1 vs. PDgr_1
*r*	−0.311^**^	−0.052	−0.029	−0.014	−0.032
	SEP2_1 vs. PDgr_1	TPL_1 vs. PDgr_1	UFO_1 vs. PDgr_1	CG1_1 vs. PDgr_1	
*r*	0.055	−0.028	−0.033	0.017	
Group C^e^	AS2_2 vs. PDgr_2	ASK2_2 vs. PDgr_2	CHR11_2 vs. PDgr_2	FIL_2 vs. PDgr_2	PLP_2 vs. PDgr_2
*r*	−0.105	−0.003	0.01	0.025	−0.400^**^
	IFL_2 vs. PDgr_2	JMJ12_2 vs. PDgr_2	KNATM_2 vs. PDgr_2	MED8_2 vs. PDgr_2	SEP1_2 vs. PDgr_2
*r*	−0.078	−0.084	−0.058	−0.018	0.072
	SEP2_2 vs. PDgr_2	TPL_2 vs. PDgr_2	UFO_2 vs. PDgr_2	CG1_2 vs. PDgr_2	
*r*	−0.066	−0.282^**^	−0.109	−0.133	

*TGs, target genes; PDgr, petalous degree*.

a *Group A indicates the correlation analyses of TGs' expression patterns and PDgr in the AH population between two environments*.

b *The expression levels of TGs in the first environment*.

c *The expression levels of TGs in the second environment. r represents the Pearson correlation coefficient*.

d *Group B indicates the correlation analyses between the TGs and PDgr in the first environment*.

e *Group C indicates the correlation analyses between the TGs and PDgr in the second environment*.

Significance levels are as follows:

** *P < 0.01*.

The correlation analyses between TG expression levels and PDgr indicated that only three TGs, *CHR11, PLP*, and *INTERFASCICULAR FIBERLESS* (*IFL*), were significantly and negatively correlated with PDgr in the first environment, while two (*PLP* and *TPL*) were significantly and negatively correlated to PDgr in the second environment (Table 1). Noticeably, based only on the correlation between TGs and PDgr, it is impossible to explain the molecular mechanism underlying the apetalous characteristic of rapeseed. In fact, the correlation analysis cannot determine the regulatory relationship between genotype and phenotype, because many genes usually participate in the regulation of phenotypic variation in an indirect manner.

### QTT-association mapping for PDgr and TG expression levels

To study relationships between PDgr and the TGs, QTT-association analyses of both PDgr and TG expression levels in the AH population were performed in two environments.

In the first environment, QTT-association analysis of PDgr indicated that *PLP* was the only QTT (−*LogP* = 9.86, *h*^2^ = 18.62%) associated with PDgr that had an obvious and negative effect on PDgr. As shown in Table 2, the effect of *PLP* on PDgr was −6.88, meaning that PDgr will be decreased 6.88% when the expression level of *PLP* increases one unit in value. The transcript-association mapping of *PLP* expression levels showed that only *CHR11* (−*LogP* = 9.08, *h*^2^ = 17.28%) was associated with *PLP* expression, and the effect was 46.77, meaning that the expression level of *PLP* would be up-regulated 46.77 units in value when that of *CHR11* was up-regulated one unit in value (Table 2). Subsequently, the QTT analysis of *CHR11* expression levels detected two tQTTs regulating *CHR11* expression, *PLP* and *JUMONJI DOMAIN-CONTAINING PROTEIN 12* (*JMJ12*) × *SEP2*, and the transcript epistasis loci *JMJ12*×*SEP2* had a negative effect on *CHR11* (Table 2). By analogy, QTT-association mapping for *JMJ12, SYMMETRIC LEAVES 2* (*AS2*), *MEDIATOR SUBUNIT 8* (*MED8*), *CG1, ARABIDOPSIS SKP1 HOMOLOGUE 2* (*ASK2*), *KNOX ARABIDOPSIS THALIANA MEINOX* (*KNATM*), *UNUSUAL FLORAL ORGANS* (*UFO*), *SEP2, FILAMENTOUS FLOWER* (*FIL*), *TPL, SEP1*, and *IFL* expression levels suggested the existence of one to six tQTTs (Table 2, Table S5). In addition to *FIL* and *SEP1*, there was at least one major tQTT (*h*^2^ ≥ 10%) for each TG. Furthermore, there was always one stable tQTT (repeatedly detected in the two environments) for eight TGs except for *CHR11, TPL*, and *SEP1*, while there are two for *CG1*. Specifically, *AS2* was a stable tQTT with a positive effect for *JMJ12*, and *JMJ12* acted as the positive and stable tQTT regulating *AS2, CG1*, and *UFO* expression. *UFO*, as a stable tQTT, played a positive role in the regulation of *CG1* and *SEP2* expressions. *KNATM* served as a stable tQTT positively regulating *ASK2* and *IFL* expressions. *ASK2*, as the stable tQTT, had positive effects on *KNATM* and *FIL* expression levels. In addition, there was at least one transcript epistasis loci for TG expression apart from *PLP, CG1, FIL, SEP1*, and *IFL* (Table S5).

**Table 2:** The key QTTs and tQTTs for PDgr and TGs detected in the first environment.

**Trait**	**QTT^a^ (tQTT)^b^**	**Effect^c^**	**Predict^d^**	***SE***	***−Logp***	***h*^2^(%)**	**EC(A-H)^e^**	**PV^f^**
Petalous degree	**PLP**	**q**	−**6.88**	**1.072**	**9.86**	**18.62**	**1464.34**	−**10079.21**
PLP expression	*CHR11*	*q*	*46.77*	*7.614*	*9.08*	*17.28*	*2.86*	*133.95*
CHR11 expression	*PLP*	*q*	*0.31*	*0.046*	*11.12*	*12.91*	*1464.34*	*457.9*
JMJ12 expression	**AS2**	**q**	**0.51**	**0.041**	**34.55**	**29.86**	−**1.09**	−**0.55**
	*MED8*	*q*	*0.34*	*0.041*	*16.13*	*13.57*	−*0.4*	−*0.14*
	*CG1*	*q*	*0.36*	*0.041*	*17.97*	*15.12*	−*73.77*	−*26.65*
AS2 expression	*ASK2*	*q*	*0.74*	*0.063*	*30.8*	*19.08*	−*2.68*	−*1.97*
	**JMJ12**	**q**	**0.87**	**0.063**	**42.21**	**26.42**	−**0.24**	−**0.21**
	*CG1*	*q*	*0.75*	*0.063*	*31.84*	*19.75*	−*73.77*	−*55.28*
MED8 expression	*JMJ12*	*q*	*0.49*	*0.046*	*26.01*	*32.35*	−*0.24*	−*0.12*
CG1 expression	*AS2*	*q*	*51.28*	*6.725*	*13.59*	*13.58*	−*1.09*	−*55.99*
	**JMJ12**	**q**	**91.27**	**6.725**	**41.04**	**43.02**	−**0.24**	−**21.67**
	**UFO**	**q**	**18.72**	**6.725**	**2.27**	**1.81**	−**21.97**	−**411.33**
ASK2 expression	**KNATM**	**q**	**2.11**	**0.185**	**29.43**	**36.26**	−**1.74**	−**3.68**
KNATM expression	**ASK2**	**q**	**1.33**	**0.125**	**25.75**	**35.47**	−**2.68**	−**3.56**
UFO expression	**JMJ12**	**q**	**32.67**	**2.47**	**39.05**	**37.32**	−**0.24**	−**7.76**
	*SEP2*	*q*	*25.74*	*2.484*	*24.36*	*23.16*	−*84.05*	−*2163.26*
SEP2 expression	**UFO**	**q**	**255.74**	**0.001**	**300**	**12.75**	−**21.97**	−**5619.2**
	*FIL × TPL*	*qq*	*−668.94*	*0.001*	*300*	*87.25*	*115.04*	−*76952.77*
FIL expression	**ASK2**	**q**	**0.26**	**0.084**	**2.75**	**4.56**	−**2.68**	−**0.7**
TPL expression	*ASK2*	*q*	*38.47*	*2.478*	*53.33*	*34.03*	−*2.68*	−*103.09*
	*ASK2 × SEP2*	*qq*	*39.91*	*5.992*	*10.55*	*36.61*	−*1088.97*	−*43455.73*
SEP1 expression	FIL	q	0.12	0.028	5.02	9.58	−0.83	−0.1
IFL expression	**KNATM**	**q**	**0.85**	**0.137**	**9.21**	**16.08**	−**1.74**	−**1.48**

a *QTT, quantitative trait transcript associated with PDgr*.

b *tQTT, QTT regulating TGs expression*.

c *q indicates the individual transcript loci, and qq indicates the additive by additive effects*.

d *Predicted effect of QTT or tQTT for the target trait. SE, standard error. −Logp, the minus log of the P-value for detecting a significant QTT or tQTT. h^2^, the heritability of QTT or tQTT*.

e Expression change of QTT or tQTT, the incremental expression level of QTT or tQTT in “APL01” compared with in “Holly.”

f *Phenotypic variation of target trait, the incremental phenotype variation in “APL01” compared with in “Holly.” The bold QTTs or tQTTs are detected repeatedly in all two environments. The italic tQTTs are detected only in the first environment*.

In the second environment, QTT-association mapping for PDgr still showed that only *PLP* (−*LogP* = 7.79, *h*^2^ = 15.11%) was negatively associated with PDgr, and the effect was −6.13 (Table 3). However, the QTT analysis of *PLP* expression levels did not detect any tQTT associated with *PLP*, implying that genes other than the 14 TGs in the present study regulate *PLP* expression. In the same way, the QTT mapping of *AS2, JMJ12, UFO, CG1, FIL, TPL, IFL, SEP2, ASK2*, and *KNATM* expression levels suggested the existence of one to five tQTTs (Table 3, Table S6). Compared with the first environment, in addition to the 10 stable tQTTs, there was also at least one major tQTT (*h*^2^ ≥ 10%) that was only detected in the second environment for the six TGs, including *PLP, JMJ12, UFO, CG1, TPL*, and *ASK2* (Table 3, Table S6). In particular, *UFO* was the major tQTT positively regulating *JMJ12* expression. *CG1*, as a major tQTT, had a positive effect on *UFO* expression. The major transcript epistasis loci, *FIL*×*TPL* and *IFL*×*SEP2*, played positive roles in the regulation of *CG1* expression. For the two major tQTTs regulating *TPL* expression, *IFL*×*SEP2* served as a positive regulator, while *KNATM*×*SEP2* acted as a negative regulator. Another major tQTT (*FIL*×*KNATM*) for *ASK2* showed a positive effect on the regulation of *ASK2* expression. Furthermore, just like in the first environment, there was a universal transcript epistatic effect among most of TGs (Table 3, Table S6), suggesting that the epistatic effect between TGs was vital regulator of TG expression. In addition, QTT analyses of *CHR11, SEP1*, and *MED8* did not detect any tQTT.

**Table 3:** The key QTTs and tQTTs for PDgr and TGs detected in the second environment.

**Trait**	**QTT^a^ (tQTT)^b^**	**Effect^c^**	**Predict^d^**	***SE***	***−Logp***	***h^2^*(%)**	**EC(A-H)^e^**	**PV^f^**
Petalous degree	**PLP**	**q**	−**6.13**	**1.084**	**7.79**	**15.11**	**1182.48**	−**7243.16**
AS2 expression	**JMJ12**	**q**	**4.76**	**0.134**	**268.05**	**72.04**	−**0.41**	−**1.94**
JMJ12 expression	**AS2**	**q**	**1.77**	**0.044**	**300**	**71.86**	−**1.87**	−**3.32**
	*UFO*	*q*	*0.79*	*0.044*	*72.05*	*14.28*	−*30.95*	−*24.48*
UFO expression	**JMJ12**	**q**	**22.7**	**1.324**	**64.64**	**42.25**	−**0.41**	−**9.24**
	*CG1*	*q*	*16.72*	*1.321*	*35.88*	*22.94*	−*90.88*	−*1519.87*
CG1 expression	**JMJ12**	**q**	**189.96**	**4.464**	**300**	**41.14**	−**0.41**	−**77.35**
	**UFO**	**q**	**30.07**	**4.452**	**10.84**	**1.03**	−**30.95**	−**930.82**
	*FIL × TPL*	*qq*	*179.13*	*11.35*	*55.04*	*36.58*	*69.61*	*12468.74*
	*IFL × SEP2*	*qq*	*120.76*	*4.267*	*172.11*	*16.63*	−*154.73*	−*18686.17*
FIL expression	**ASK2**	**q**	**1.58**	**0.139**	**29.27**	**42.27**	−**2.08**	−**3.29**
TPL expression	*IFL × SEP2*	*qq*	*1515.97*	*0*	*300*	*80.2*	−*154.73*	−*234570.01*
	*KNATM × SEP2*	*qq*	*−753.16*	*0*	*300*	*19.8*	−*25.6*	*19279.63*
IFL expression	**KNATM**	**q**	**41.38**	**4.531**	**19.14**	**28.74**	−**1.49**	−**61.48**
	CG1	q	24.36	4.518	7.15	9.96	−90.88	−2213.99
SEP2 expression	**UFO**	**q**	**30.13**	**4.383**	**11.19**	**21.27**	−**30.95**	−**932.64**
ASK2 expression	**KNATM**	**q**	**6.63**	**0.309**	**100.36**	**62.65**	−**1.49**	−**9.84**
	*FIL × KNATM*	*qq*	*2.69*	*0.137*	*84.23*	*10.35*	−*8.07*	−*21.74*
KNATM expression	**ASK2**	**q**	**7.67**	**0.223**	**252.36**	**85.14**	−**2.08**	−**15.95**

*The definitions of a–f are the same as in Table 2. The bold QTTs or tQTTs are detected repeatedly in all two environments. The italic tQTTs are detected only in the second environment*.

### Unconditional and conditional eQTL mapping of TGs

In addition to aforementioned tQTTs, the genomic region is another key factor influencing TG expression levels. In our previous work (Wang et al., 2015), the AH map, a high-density genetic linkage map of 2,027.53 cM with an average marker interval of 0.72 cM, has been constructed and used to identify QTLs for PDgr. An eQTL analysis for TGs was performed based on the AH map.

**Figure 3:**
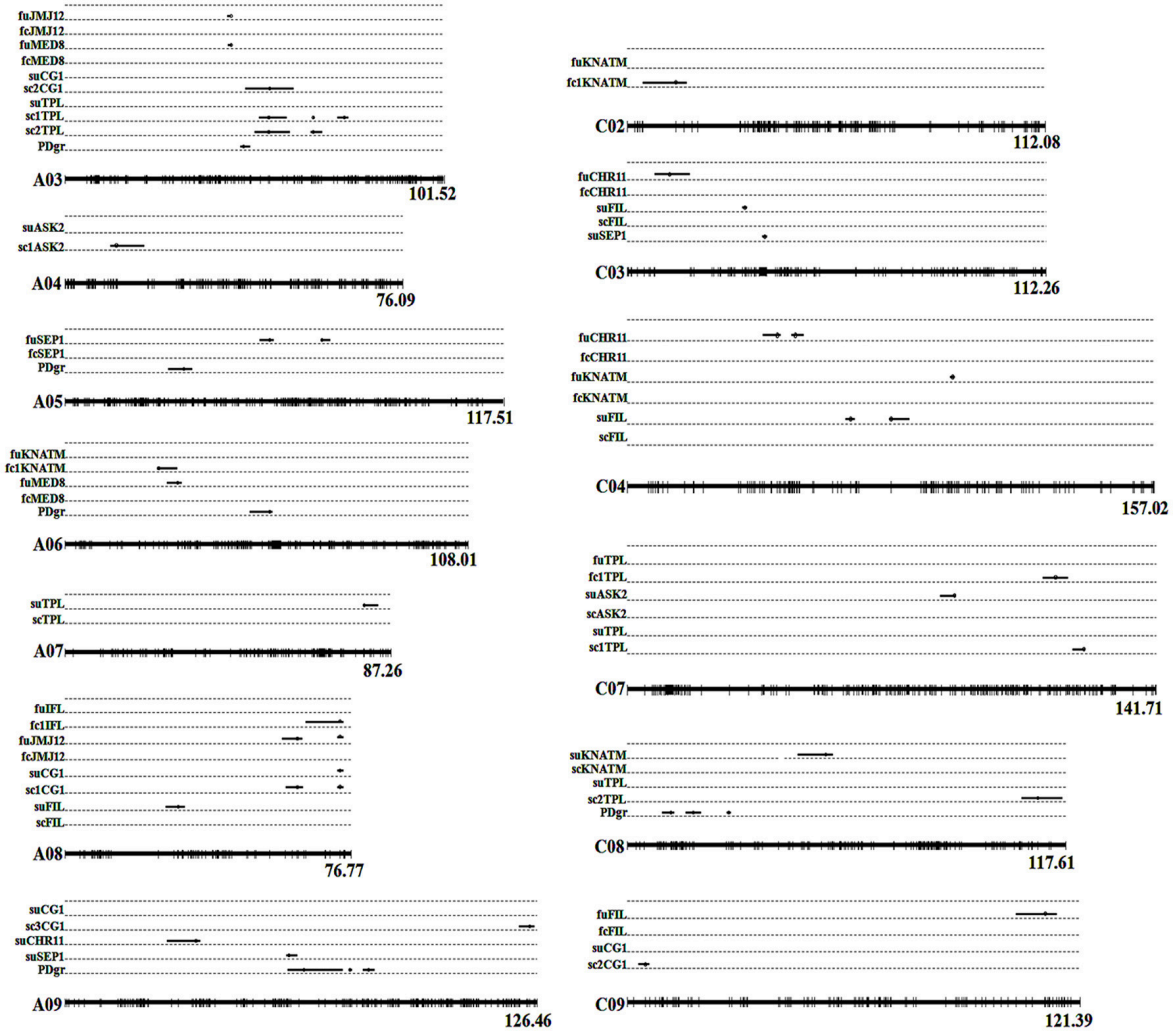
Alignments between unconditional and conditional eQTLs of TG expression levels in two environments. Whole linkage groups are represented by *black lines* labeled with molecular markers (*short vertical bars*) on the bottom. The *Arabic numerals* listed on the right side indicate the lengths of the linkage groups. The TGs' unconditional and conditional expression levels are listed on the left side. “fu” represents the TG's unconditional expression level, while “fc” represents the TG's conditional expression level in the first environment. “su” represents the TG's unconditional expression level, while “sc” represents the TG's conditional expression level in the second environment. The black lines on the linkage groups show the QTL confidence interval and the circles indicate the peak position. Detailed information of eQTLs is shown in Tables 4, 5. PDgr is the acronym of petalous degree. fcCHR11: *CHR11|PLP, CHR11|MED8*; fcFIL: *FIL|ASK2, FIL|SEP1*; fc1IFL: *IFL|ASK2*; fcKNATM: *KNATM|ASK2, KNATM|IFL*; fcJMJ12: *JMJ12|AS2, JMJ12|MED8, JMJ12|CG1*; fc1KNATM: *KNATM|IFL*; fcMED8: *MED8|CHR11, MED8|JMJ12*; fcSEP1: *SEP1|FIL*; fc1TPL: *TPL|ASK2*×*SEP2*. scASK2: *ASK2|KANT, ASK2|FILxKNATM*; sc1ASK2: *ASK2|FIL*×*KNATM*; scCG1: *CG1|JMJ12, CG1|UFO, CG1|FIL*×*TPL, CG1|IFL*×*SEP2*; sc1CG1: *CG1|FIL*×*TPL*; sc2CG1: *CG1|IFL*×*SEP2*; sc3CG1: *CG1|UFO*; scFIL: *FIL|ASK2*; scKNATM: *KNATM|ASK2*; scTPL: *TPL|IFL*×*SEP2, TPL|KNATM*×*SEP2*; sc1TPL: *TPL|IFL*×*SEP2*; sc2TPL: *TPL|KNATM*×*SEP2*.

In the current study, unconditional eQTL linkage mapping of 14 TG expression levels in the first environment suggested the existence of one to three eQTLs (Figure 3, Table 4, Table S7), and *uqCHR11C4-2, uqSEP1A5-1*, and *uqSEP1A5-1* explained 11.17, 10.76, and 10.11%, respectively, of the estimated phenotypic variation, while the remaining eQTLs explain less than 10% (Table 4, Table S7). Further analyses of the eQTLs determined that *uqJMJ12A3* (43.5–44.4 cM) shared the same single-nucleotide polymorphism marker (Bn-A03-p15435174, 44.42 cM) with *uqMED8A3* (43.6–44.4 cM), and the two eQTLs were close to *qPD.A3* (46.9–49.5 cM) for PDgr (Wang et al., 2015), and may be regarded as pleiotropic effects caused by the same locus. However, none of the unconditional eQTLs colocalized with the QTLs identified in the previous study for PDgr (Figure 3, Table S7). Furthermore, all of the unconditional eQTLs mapped to chromosomes different from the corresponding TGs, which means that these eQTLs are trans-acting factors based on the classification rules of eQTL (Kliebenstein, 2008; Sasayama et al., 2012). To evaluate the reliability of QTT analysis results in the first environment, a conditional eQTL analysis was carried out as described by Zhu (1995). Because there is almost one key tQTT (*h*^2^ ≥ 10% or repeatedly detected in the two environments) for each TG, their conditional expression levels for the key tQTT can be generated using the QGAstation software. Conditional eQTL mapping suggested that only four conditional eQTLs, *cqIFLA8, cqKNATMA6, cqKNATMC2*, and *cqTPLC7*, were obtained (Table 4), and they were different from the unconditional eQTLs (Figure 3). The result suggested that the four conditional eQTLs were suppressed by the corresponding conditional independent variables, *ASK2, IFL*, and *ASK2*×*SEP2*, under the unconditional situation. Furthermore, the four conditional eQTLs had negative effects on the corresponding TGs expression, which implied that *ASK2, IFL*, and *ASK2*×*SEP2* could act as the positive regulator of *IFL, KNATM*, and *TPL* expression. Interestingly, the results were consistent with the results of QTT analyses. Thus, conditional eQTL analyses further confirm the validity of QTT-association mapping for TGs expression levels.

**Table 4:** The eQTLs for TGs unconditional and conditional expression levels in the first environment.

**Trait**	**eQTL**	**Chr^a^**	**Peak**	**Marker^b^**	**CI^c^**	***LOD***	***R^2^*(%)**	***Add*^d^**	**Env.^e^**	**Acting^f^**
CHR11 expression	*uqCHR11C3*^g^	C3	11.41	Bn-scaff_16614_1-p546020 (7.382)	7.4–16.8	4.08	9.61	−0.26	2015a	trans-eQTL
	*uqCHR11C4-1*	C4	44.61	Bn-scaff_15908_1-p289000 (44.593)	40.3–45.5	3.70	7.63	−0.23	2015a	trans-eQTL
	*uqCHR11C4-2*	C4	50.01	Bn-scaff_19248_1-p207144 (49.936)	48.9–52.5	5.59	11.17	−0.28	2015a	trans-eQTL
FIL expression	*uqFILC9*	C9	112.01	Bn-scaff_17750_1-p587349 (111.994)	104.2–115.1	3.62	5.66	−0.30	2015a	trans-eQTL
JMJ12 expression	***uqJMJ12A3***	**A3**	**44.41**	**Bn-A03-p15435174 (44.42)**	**43.5**–**44.4**	**3.02**	**6.02**	**0.41**	**2015a**	**trans-eQTL**
	*uqJMJ12A8-1*	A8	62.31	Bn-A08-p19642040 (62.239)	58.2–63.5	3.11	6.81	0.54	2015a	trans-eQTL
	*uqJMJ12A8-2*	A8	73.71	Bn-A08-p20855631 (73.676)	73.1–74.7	3.74	7.59	−0.57	2015a	trans-eQTL
KNATM expression	*uqKNATMC4*	C4	96.91	BRAS021 (96.859)	96.2–97.5	3.99	8.84	−0.83	2015a	trans-eQTL
MED8 expression	***uqMED8A3***	**A3**	**44.41**	**Bn-A03-p15435174 (44.42)**	**43.6**–**44.4**	**2.94**	**6.43**	**0.27**	**2015a**	**trans-eQTL**
	*uqMED8A6*	A6	30.21	Bn-A06-p4933932 (30.205)	27.4–31.3	3.34	7.39	0.27	2015a	trans-eQTL
SEP1 expression	*uqSEP1A5-1*	A5	54.81	BnGMS294 (56.076)	52.1–55.8	3.75	10.76	−0.16	2015a	trans-eQTL
	*uqSEP1A5-2*	A5	68.81	Bn-A05-p17758084 (68.739)	68.5–71.0	4.46	10.11	0.16	2015a	trans-eQTL
IFL|ASK2 expression	*cqIFLA8*^h^	A8	73.71	Bn-A08-p20855631 (73.676)	64.6–74.7	3.24	7.01	−0.52	2015a	trans-eQTL
KNATM|IFL expression	*cqKNATMA6*	A6	25.11	Bn-A06-p4056446 (25.131)	24.9–30.1	3.75	8.10	−0.65	2015a	trans-eQTL
	*cqKNATMC2*	C2	13.01	Bn-scaff_15714_1-p1091353 (12.977)	4.2–15.9	3.58	7.66	−0.63	2015a	trans-eQTL
TPL|ASK2 × SEP2 expression	*cqTPLC7*	C7	114.71	Bn-scaff_16069_1-p4085872 (114.718)	111.3–118.0	3.29	7.28	−12.01	2015a	trans-eQTL

a *Chromosome*.

b *The closest marker and the marker position in the AH map*.

c *The 2-LOD confidence interval of eQTLs*.

d *Additive effects*.

e *2015a represents the first environment in which the eQTLs were detected*.

f *The classification of eQTLs, cis-eQTL is mapped to the same genomic location like an expressed gene (within 5 Mb) while trans-eQTL is mapped to a different genomic location from an expressed gene (>5 Mb or on different chromosomes)*.

g *The unconditional eQTLs of TGs expression levels*.

h *The conditional eQTLs of TGs conditional expression levels. The bold eQTLs are at the same position with each other*.

In the second environment, unconditional eQTL analyses of the 10 TGs showed that only 11 unconditional eQTLs for 7 TGs were detected (Figure 3, Table 5, and Table S8). All of the unconditional eQTLs were distinguishable from those detected in the first environment (Figure 3). Comparing to QTLs identified for PDgr in a previous study, the confidence interval of *uqSEP1A9* (59.4–62.2 cM) overlapped that of *qPD.A9-1* (59.66–74.36 cM) (Figure 3, Table S8), suggested that *qPD.A9-1* participates in the petal development of line APL01 by regulating *SEP1* expression. In the relationship between the unconditional eQTL and the corresponding TG, *uqTPLA7* is a cis-acting factor (within 5 Mb), while the remaining 10 unconditional eQTLs are trans-acting factors (on different chromosomes). In addition, just as in the first environment, the conditional expression levels of the TGs were obtained using the key tQTTs. The conditional eQTL mapping of TGs showed that 13 conditional eQTLs for 6 TGs were obtained (Figure 3, Table 5). The conditional eQTL *uqCG1A8* (73.1–74.7 cM) is the same as the unconditional eQTL *cqCG1A8-2* (73.1–74.7 cM), while the remaining conditional eQTLs are novel compared with the unconditional eQTLs (Figure 3, Table 5). Over half conditional eQTLs had negative effects on the corresponding TGs, which was consistent with the QTT mapping results. More detailed information on the conditional eQTLs was provided in Table 5 and Table S8.

**Table 5:** The eQTLs for TGs unconditional and conditional expression levels in the second environment.

**Trait**	**eQTL**	**Chr^a^**	**Peak**	**Marker^b^**	**CI^c^**	***LOD***	***R^2^*(%)**	***Add*^d^**	**Env.^e^**	**Acting^f^**
ASK2 expression	*qASK2C7*^g^	C7	87.61	Bn-scaff_15705_1-p2283255 (87.581)	83.8–87.9	2.56	5.74	−3.46	2015b	trans-eQTL
CHR11 expression	*qCHR11A9*	A9	35.11	Bn-A09-p17415894 (35.144)	27.4–36.2	2.75	7.01	−4.05	2015b	trans-eQTL
FIL expression	*qFILA8*	A8	30.41	Bn-A08-p15723645 (30.366)	27.0–32.1	3.68	7.34	−0.68	2015b	trans-eQTL
FIL expression	*qFILC3*	C3	31.61	Bn-scaff_22728_1-p1065288 (31.571)	30.9–32.1	3.42	6.98	0.66	2015b	trans-eQTL
FIL expression	*qFILC4-1*	C4	66.51	Bn-scaff_20042_1-p1582 (66.539)	65.0–67.7	7.70	16.48	1.29	2015b	trans-eQTL
FIL expression	*qFILC4-2*	C4	78.61	Bn-scaff_26946_1-p121318 (78.628)	78.0–84.0	4.77	10.15	−1.00	2015b	trans-eQTL
KNATM expression	*qKNATMC8*	C8	53.21	Bn-scaff_16770_1-p3966893 (53.829)	45.7–55.0	2.97	6.70	−2.20	2015b	trans-eQTL
SEP1 expression	***qSEP1A9***^i^	**A9**	**60.01**	**Bn-A09-p25821544 (59.96)**	**59.4**–**62.2**	**3.01**	**7.03**	−**1.10**	**2015b**	**trans-eQTL**
SEP1 expression	*qSEP1C3*	C3	36.81	Bn-scaff_18322_1-p2155092 (36.875)	36.5–37.0	2.63	5.78	0.75	2015b	trans-eQTL
TPL expression	*qTPLA7*	A7	80.11	Bn-scaff_25466_1-p15589 (80.094)	79.9–83.7	2.54	3.97	334.00	2015b	cis-eQTL
CG1 expression	***qCG1A8***^j^	***A8***	***73.71***	***Bn-A08-p20855631 (73.676)***	***73.1***–***74.7***	***3.37***	***7.02***	–***80.35***	***2015b***	***trans-eQTL***
ASK2|FIL × KNATM expression	*cqASK2A4*^h^	A4	11.61	Bn-A04-p3820381 (11.625)	10.2–17.8	3.07	7.17	1.23	2015b	trans-eQTL
CG1|FIL × TPL expression	*cqCG1A8-1*	A8	62.31	Bn-A08-p19642040 (62.239)	59.3–63.8	3.31	7.44	80.14	2015b	trans-eQTL
	***qCG1A8-2***^j^	***A8***	***73.71***	***Bn-A08-p20855631 (73.676)***	***73.1***–***74.7***	***3.45***	***7.23***	–***79.27***	***2015b***	***trans-eQTL***
CG1|IFL × SEP2 expression	*cqCG1A3*	A3	54.81	Bn-scaff_17298_1-p705887 (54.794)	48.2–61.0	3.24	8.37	63.50	2015b	trans-eQTL
	*cqCG1C9*	C9	4.81	CB10103 (4.82)	3.0–5.8	4.58	12.26	79.61	2015b	trans-eQTL
CG1|UFO expression	*cqCG1A9*	A9	124.51	Bn-scaff_16389_1-p578073 (124.527)	121.7–125.8	3.65	8.32	37.00	2015b	trans-eQTL
TPL|IFL × SEP2 expression	*cqTPLA3-1*	A3	54.51	Bn-A03-p16431100 (54.482)	52.0–59.3	6.12	16.99	−477.35	2015b	trans-eQTL
	*cqTPLC7*	C7	122.31	Bn-scaff_16110_1-p3700752 (122.401)	119.3–122.6	3.10	6.97	−228.88	2015b	trans-eQTL
TPL|KNATM × SEP2 expression	*cqTPLA3-1*	A3	54.51	Bn-A03-p16431100 (54.482)	50.7–60.0	5.27	14.28	−556.66	2015b	trans-eQTL

*The definitions of a–d and f-h are the same as in Table 4*.

e *2015b represents the second environment in which the eQTLs were detected*.

i *The eQTL is at the same approximate position with qPD.A9-1 identified in the previous study for PDgr*.

j *The eQTLs are at the same position with each other*.

### TGs regulate petal development through *CHR11-PLP* pathway

**Figure 4:**
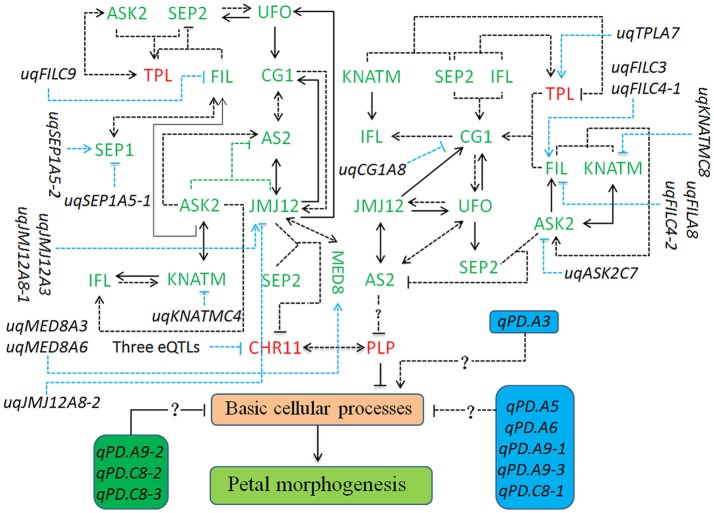
The regulatory network involved in the petal development of apetalous “APL01.” The regulatory network mainly consists of the *CHR11-PLP* pathway, 12 unconditional eQTLs of TGs, and nine QTLs for PDgr. The *CHR11-PLP* pathway contains 29 tQTTs and 12 unconditional eQTLs, representing 41 kinds of regulatory relationships. The three eQTLs negatively regulating *CHR11* expression are *uqCHR11C3, uqCHR11C4-1*, and *uqCHR11C4-2*. Genes marked in red are up-regulated, while genes marked in green are down-regulated in “APL01” compared with those in “Holly.” Arrows represent the positive regulation of tQTTs for the downstream TGs, while blunted lines represent the negative regulation of tQTTs for the downstream TGs. Arrows or blunted solid lines marked indicate the regulatory relationships repeatedly detected in all two environments, while arrows or blunted dotted lines indicate the regulatory relationships only detected in one environment. In addition, there may be the *AS2-PLP* pathway regulating *PLP* expression, and this pathway consists of 21 tQTTs and 8 unconditional eQTLs, representing 29 kinds of regulatory relationships.

Based on the QTTs and unconditional eQTLs in this study, together with our previous works (Wang et al., 2015; Yu et al., 2016), a hypothetical regulatory network involved in petal development of “APL01” was constructed. As shown in Figure 4, the 14 petal regulators potentially regulate the petal development of “APL01” through the *CHR11*-*PLP* pathway. *PLP* acts as the terminal signal integrator negatively regulating petal development in the *CHR11-PLP* pathway. In addition, *PLP* expression level may be negatively regulated by *AS2* in other manners as well.

The *CHR11*-*PLP* pathway consists of 29 tQTTs and 12 unconditional eQTLs (Figure 4). *PLP* directly and negatively regulates petal development of line APL01 in the *CHR11*-*PLP* pathway. *CHR11* acts as the main promoter of *PLP* expression, while *CHR11* is positively regulated by *PLP* as well. The transcripts of the epistatic loci *JMJ12*×*SEP2* are key negative regulator of *CHR11*. Three unconditional eQTLs with negative effects, *uqCHR11C3, uqCHR11C4-1*, and *uqCHR11C4-2*, also participate in the regulation of *CHR11* expression. For the *JMJ12* expression level, there are two positive closed regulatory circuits, in which *JMJ12*–*CG1*–*AS2*–*JMJ12* is a bidirectional circuit while *JMJ12*–*UFO*–*CG1*–*AS2*–*JMJ12* is a unidirectional circuit. Moreover, two unconditional eQTLs (*uqJMJ12A3* and *uqJMJ12A8-1*) with positive effects and the repressive *uqJMJ12A8-2* also participated in the regulation of *JMJ12* expression. Additionally, *JMJ12* positively regulates *MED8*. In the *JMJ12*–*CG1*–*AS2*–*JMJ12* circuit, *AS2* was also regulated by the promoter *ASK2*, and the transcript epistatic loci (*ASK2*×*JMJ12*) had a negative effect. In addition, *ASK2* was positively regulated by *ASK2*–*KNATM*–*IFL*–*ASK2*, a unidirectional circuit. In the *JMJ12*–*UFO*–*CG1*–*AS2*–*JMJ12* circuit, the *UFO* expression level was also regulated by the promoter *SEP2*, while *SEP2* expression level was attributed to the integrated regulation of the promoter *UFO* and the transcripts of the epistatic loci (*FIL*×*TPL*) had a negative effect. Furthermore, *FIL* was regulated by both activators (*ASK2* and *SEP1*) and the repressive *uqFILC9*, while *TPL* was positively regulated by both the activator *ASK2* and the transcript epistatic loci (*ASK2*×*SEP2*), which had a positive effect. Finally, the regulatory effects of the tQTTs and unconditional eQTLs were integrated into the expression level of *PLP* and then prevented the basic cellular processes responsible for petal morphogenesis by up-regulating *PLP* (Figure 4).

In addition to *CHR11, PLP* expression level may be also regulated by the suppressor *AS2* (Figure 4). However, the regulation of *PLP* by *AS2* probably requires gene other than the above 14 petal regulators. The *AS2* expression level was attributed to the integrated regulation of multi-factors containing 21 tQTTs and 8 unconditional eQTLs (Figure 4).

## Discussion

There are a large number of upstream regulators involved in petal development in *Arabidopsis* (Zik and Irish, 2003; Kaufmann et al., 2009, 2010; Wuest et al., 2012). In a previous study (Yu et al., 2016), 36 petal regulators and several candidate genes involved in the regulation of the apetalous trait in *B. napus* were identified. However, how these genes collaboratively regulate petal development in both *Arabidopsis* and *B. napus* is unclear. In this study, we determined that 14 TGs participate in the regulation of apetalous characteristic in “APL01” by analyzing the expression patterns of 37 petal regulators in “APL01,” “PL01,” and “Holly.” The same slopes of the standard curvesof 14 TGs and the endogenous reference gene *ACTIN* indicated the same amplification efficiency. Thus, the use of qRT-PCR in the AH population is dependable (Yin et al., 2010).

From the Pearson correlation coefficients, the similarity level of PDgr in the AH population is high between the two environments (*r* = 0.806) but not completely the same, which can probably be ascribed to unknown environmental effects (Wang et al., 2015). The similarities of the TG expression patterns in the AH population are poor between the two environments (*r* < 0.8), except for five TGs, which congruously explains the variation in PDgr between the two environments. The correlation analyses between the 14 TGs and PDgr determined that only a few TGs were significantly correlated (*P* < 0.05) with PDgr in the two environments, implying that only a few genes were directly related to petal development. In fact, several previous researches have suggested that many transcriptional regulators indirectly regulate petal development in one way or another (Zik and Irish, 2003; Kaufmann et al., 2009, 2010; Wuest et al., 2012). However, a linear correlation analysis failed to discover the intricate relationships between genes and petal morphogenesis.

QTT-association mapping, based on a mixed linear model, is mainly used to analyze complex traits (Zhang et al., 2015). A QTT analysis of PDgr determined that *PLP* acts as the major negative QTT of PDgr in the two environments, indicating that *PLP* negatively regulates petal development in *B. napus*. In *Arabidopsis, PLP* encodes the alpha-subunit shared between protein farnesyltransferase and protein geranylgeranyltransferase-I (Running et al., 2004). *plp* mutant leads to dramatically enlarged meristems and increased floral organ number (Running et al., 2004). Based on the high degree of chromosomal colinearity between *B. napus* and *Arabidopsis* (Chalhoub et al., 2014), it is very likely that *BnPLP* plays the same role in regulating petal development as *AtPLP*. Except for *PLP*, the remaining TGs were not significantly associated with PDgr, suggesting that these TGs potentially participate in petal development of rapeseed by regulating *PLP* expression.

The QTT mapping of *PLP* expression levels showed that *CHR11* was positively associated with *PLP* in the first environment, indicating that *CHR11* acts as a positive regulator of *PLP* expression. However, we can not detect the effect of *CHR11* on *PLP* in the second environment, implying that *CHR11* regulates *PLP* expression in an environment dependent way. Previous reports suggested that *CHR11* encoded a SWI2/SNF2 chromatin remodeling protein belonging to the ISWI family that was involved in the epigenetic regulation of eukaryotic genes (Li et al., 2012, 2015). In the second environment, the effect of *CHR11* on *PLP* may be too weak to detect by QTT-association mapping because of some unqualified environmental conditions. By analogy, QTT mapping for the remaining TGs detected 38 tQTTs, associated with 13 TGs, and 31 tQTTs, associated with 10 TGs in the first and second environment, respectively. A total of 10 tQTTs can be repeatedly detected in the two environments, implying that these regulatory relationships may occur *in vivo*, as well as being required for petal development in *B. napus*. In addition, the detection of some tQTTs in one environment might be the result of the different expression patterns of TGs between two environments. Meanwhile, these tQTTs may act as the decisive factors that give rise to variable PDgr between the two environments because gene expression' diversity is a vital mechanism underlying phenotypic diversity among individuals (Yin et al., 2010). Thus, the different tQTTs between the two environments are also required for petal development.

For the molecular functions of QTT or tQTT, *PLP, CHR11*, and *FIL*×*TPL*, respectively, acted as a repressor of PDgr, an activator of *PLP*, and a repressor of *SEP2* in the first environment, which echoes previous studies in *Arabidopsis* that suggested that *PLP* (Running et al., 2004), *CHR11* (Li et al., 2012) and *TPL* (Krogan et al., 2012) acted as repressors regulating petal development. There are mostly positive regulatory relationships between the remaining 10 TGs in the first environment, which supports our recent inference that the 10 TGs play positive roles in petal development in *B. napus* (Yu et al., 2016). In the second environment, the regulatory signals of the tQTTs are finally integrated into the expression level of *AS2* and may have then negatively regulated *PLP* expression by regulating some intermediate regulators (Figure 4); however, we cannot detect the negative effect of *AS2* on *PLP* because only a limited number of genes are included in the present study. Although the regulatory relationships among TGs presented in this study need to be verified through more molecular experiments, these relationships are logically possible. For example, *UFO*, as an essential component of the SCF complex that is a key ubiquitin E3 ligase (Skowyra et al., 1997), is involved in both floral meristem and floral organ development in *Arabidopsis* (Levin and Meyerowitz, 1995). In this study, *UFO* probably regulates the expression of *SEP2, CG1*, and *JMJ12* in a LEAFY-dependent manner, just like it regulates *AP3* transcription in *Arabidopsis* (Chae et al., 2008). Moreover, *CG1* as a candidate gene in the CI of *qPD.C8-2* regulating the apetalous trait in line APL01 functions upstream of the *CHR11-PLP* pathway, implying that *qPD.C8-2* potentially regulates the petal development of line APL01 through the *CHR11-PLP* pathway.

Unconditional eQTL mapping of TG expression levels in the AH population determined that only a few unconditional eQTLs were obtained for the TGs in two environments, and that all of the unconditional eQTLs were minor QTLs (*R*^2^ < 20%) (Shi et al., 2009). Thus, the strength of TG expression levels was mainly ascribed to effects of tQTTs. Based on the description for trans-eQTLs (Kliebenstein, 2008; Sasayama et al., 2012), all of the unconditional eQTLs presently identified are trans-eQTL, except for *uqTPLA7*, which indicates that most of the unconditional eQTLs act as transcription factors or transcriptional coactivators of the corresponding TGs. *uqSEP1A9*, a trans-eQTL identified in the second environment, overlapped a QTL (*qPD.A9-1*) for PDgr (Wang et al., 2015), indicating that the PDgr and *SEP1* expression were causally related (Thumma et al., 2001) and that the *qPD.A9-1* potentially participated in the regulation of PDgr by regulating *SEP1* expression. Furthermore, the colocalization of TG expression levels may reflect the pleiotropism of a genomic region (QTL), such as *JMJ12* and *MED8* in the first assay.

In addition, conditional eQTL mapping of TGs determined that the unconditional eQTLs were lost, except for *uqCG1A8* (*cqCG1A8-2*), in the two environments, implying that the effects of those unconditional eQTLs were completely attributed to the upstream tQTTs regarded as the conditional independent variables (Zhu, 1995). In other words, the effects of those unconditional eQTLs were passed from tQTTs to the corresponding downstream TGs, indicating that there is a relationship between the tQTT and the corresponding downstream TG, indirectly verifying the likelihood of tQTTs regulating TGs' expression levels (Zhu, 1995). Compared with the unconditional eQTLs, almost all of the conditional eQTLs are novel, indicating that these conditional eQTLs are generally suppressed by the corresponding upstream tQTTs under an unconditional situation (Zhu, 1995). That is, the upstream tQTTs participate in the positive regulation of TG expression by repressing the corresponding conditional eQTLs (Zhu, 1995). Thus, the conditional eQTLs should have negative effects on the TGs' expression, which is consistent with the results of conditional eQTL mapping in this study. Unconditional eQTL coupled with conditional QTL mapping indirectly verifies that the tQTTs detected in this study are valid.

The relationships among TGs and PDgr are presented in Figure 4. The apetalous characteristic of “APL01” is not only attributed to the regulators identified in this study, but it is possible that the aforementioned TGs participate in the petal development of “APL01” in the manner described in Figure 4. Although these regulatory relationships need to be further verified, our findings provided a basis for solving the puzzle of petal development in *B. napus*.

## Author contributions

KY and XW co-wrote the first draft of the manuscript. JZ and RG designed the project, acquired funding, and finalized the manuscript. KY and SC collected the young inflorescences of the AH population. KY and XW performed the qRT-PCR assays. QP and FC performed total RNA exaction. HL and WZ performed the first strand cDNA synthesis. SF and MH collected and processed the data used in this study. WL and PC assisted and analyzed the data. All authors have reviewed and approved the final version of the manuscript and therefore are equally responsible for the integrity and accuracy of its content.

### Conflict of interest statement

The authors declare that the research was conducted in the absence of any commercial or financial relationships that could be construed as a potential conflict of interest.

## Supplementary material

Figure S1 The scatter plot diagram of the normalized expression levels of TGs across the AH population in two environments.

Table S1 List of the primers used for qRT-PCR assays in this study.

Table S2 The expression patterns of 37 petal regulators in RNA-seq and qRT-PCR assays.

Table S3 The regression formulas of 14 TGs' amplification reactions in the standard cDNA.

Table S4 Correlation analysis between two biological replicates of TG expression within an environment.

Table S5 Highly significant QTTs and tQTTs for PDgr and TGs detected in the first environment.

Table S6 Highly significant QTTs and tQTTs for PDgr and TGs detected in the second environment.

Table S7 Comparison between QTLs for PDgr and eQTLs for TGs unconditional and conditional expression levels in the first environment.

Table S8 Comparison between QTLs for PDgr and eQTLs for TGs unconditional and conditional expression levels in the second environment.
