# Supplementary figures and images for: Quantitative Trait Transcripts Mapping Coupled with Expression Quantitative Trait Loci Mapping Reveal the Molecular Network Regulating the Apetalous Characteristic in Brassica napus L
Source: Front Plant Sci. 2018 Feb 1;9:89. doi: 10.3389/fpls.2018.00089 (PMC5810251; doi:10.3389/fpls.2018.00089)

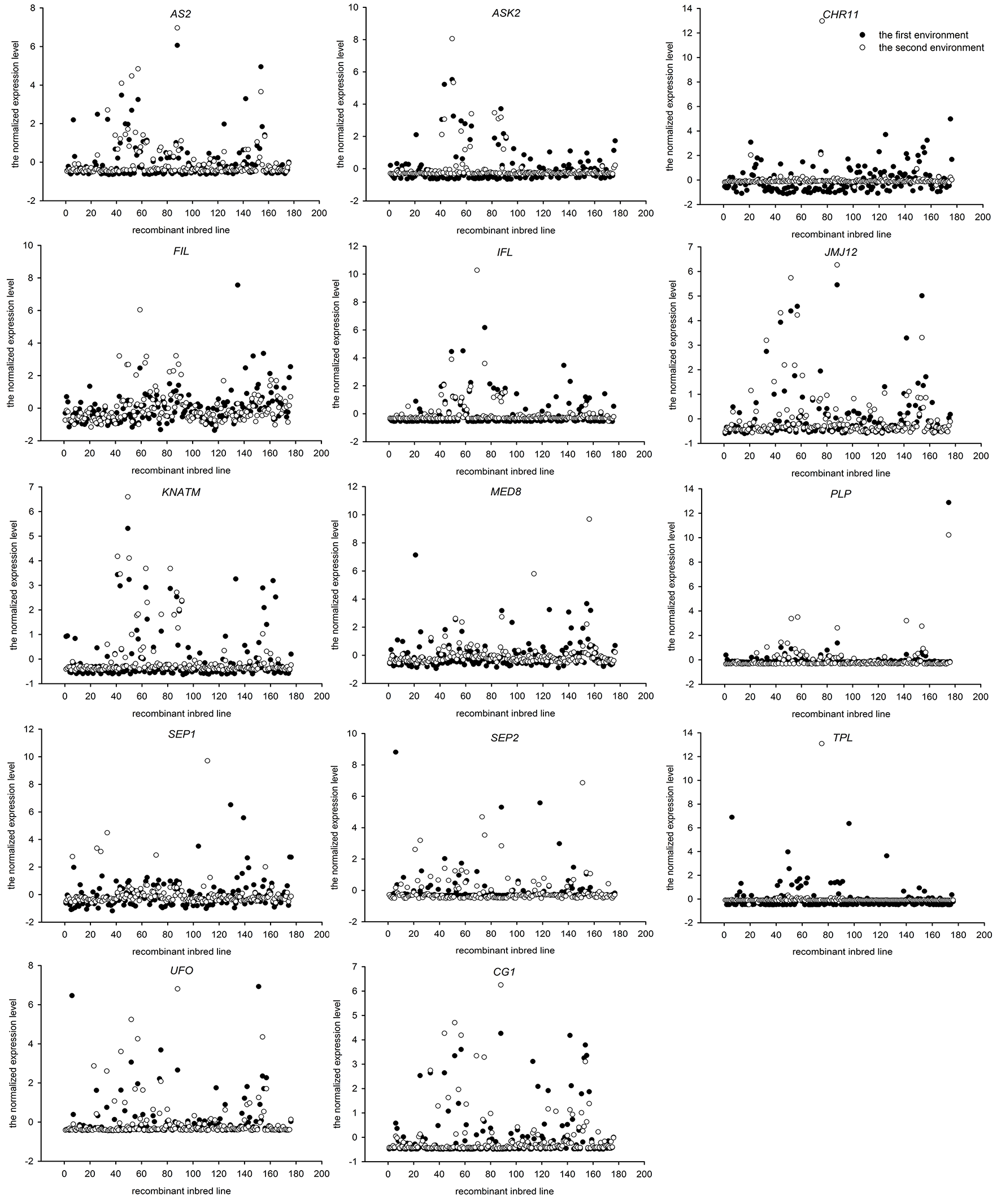

Supplement: Figure S1 — The scatter plot diagram of the normalized expression levels of TGs across the AH population in two environments. [file Image1.TIF]
